# Differential Sensitivity of Photosynthetic Electron Transport to Dark-Induced Senescence in Wheat Flag Leaves

**DOI:** 10.1186/s12870-025-06624-5

**Published:** 2025-05-16

**Authors:** Cheng Yang, Simeng Du, Yanhua Shi, Deqi Zhang, Junqin Yue, Xiangdong Li, Haiyang Jin, Baoting Fang, Fang Wei, Zishan Zhang, Ge Yan

**Affiliations:** 1 Wheat Research Institute, Henan Academy of Agricultural Sciences, Postgraduate T&R Base of Zhengzhou University, Zhengzhou, 450002 China; 2 School of Agricultural Sciences, Zhengzhou University, Zhengzhou, 450002 China; 3 State Key Laboratory of Crop Biology, College of Life Sciences, Shandong Agricultural University, Tai’an, 271018 China

**Keywords:** Wheat, Senescence, Prompt fluorescence, Modulated 820-nm reflection, Delayed fluorescence

## Abstract

**Background:**

In winter wheat (*Triticum aestivum*), delayed senescence of the flag leaf is linked to the duration of photosynthesis and grain yield. In different wheat cultivars, various components of the photosynthetic apparatus may display differences during senescence. Furthermore, previous studies related to senescence mostly used a limited number of cultivars, making it difficult to investigate the patterns and reasons for different appearance of damage to electron transport among various cultivars.To tackle these challenges, flag leaves of 32 wheat cultivars were subjected to darkness in vitro to simulate the senescence process. The cultivars were divided into three groups by k-means clustering, based on the rate of decline in their leaf chlorophyll content. Subsequently, we simultaneously measured prompt chlorophyll a fluorescence, delayed chlorophyll a fluorescence, and modulated 820-nm light reflection to examine the alterations in photosynthetic electron transport within the three groups of wheat cultivars during dark-induced senescence.

**Results:**

The results showed that the photosystem II (PSII) donor side, grouping of PSII units, PSII reaction center, PSII acceptor side, and photosystem I (PSI) were all damaged during dark-induced senescence, while the sensitivity of photosynthetic electron transport to senescence gradually increased from the upstream to downstream electron carriers on the PSII acceptor side. The extent of the observed decrease in activity of the different components of the photosynthetic electron transport chain during senescence, was consistent with the chlorophyll degradation rate of the wheat cultivars, while the priority of inhibition for different photosynthetic electron transport processes in each cultivar group was different. The results from the three separate signals align well with each other.

**Conclusions:**

The sensitivity of different part of photosynthetic electron transport to senescence were varied depended on their chlorophyll degradation rate. The differences in the response of different processes of photosynthetic electron transport to chlorophyll degradation rates might be an important factor influencing the differences in photoinhibition among wheat cultivars, especially in senescence process.

**Supplementary Information:**

The online version contains supplementary material available at 10.1186/s12870-025-06624-5.

## Introduction

To meet the demands of fast-growing population, global crop production must be doubled by 2050 [1, 2]. Increasing wheat (*Triticum aestivum*) yields is one of the most important means of achieving this goal. China has a population of more than 1.3 billion people and cultivates more than 22 million hectares of wheat annually. Food security in China and the global food supply therefore rely on stable and high wheat yields.

Plants gain the energy they need for growth and development from photosynthesis. The flag leaf is an important photosynthetic organ in the latter stages of wheat development. The sucrose produced by the flag leaf is transported to the reproductive organs, which is vital for the normal development of the anthers [3]. After flowering, wheat enters the grain-filling stage, and the flag leaf gradually starts to senesce. The initiation and progression of leaf senescence are regulated by a variety of internal and external factors such as age, phytohormones, epigenetic modifications and environmental stresses [4–6]. A recent study found that the remobilization of nitrogen from vegetative parts to grains initiates leaf senescence and is closely correlated with autophagy, which were demonstrated by the fact that N application significantly increased the N remobilization rate, delayed flag leaf senescence, and decreased in the in the expression of autophagy-related genes [7]. During senescence, the photosynthetic performance decreases, chlorophyll degrades, and the nitrogen from the flag leaf moves to the grains [8], contributing about 18% of the total grain N content [9]. The carbon assimilates produced by photosynthesis in the flag leaf were originally believed to contribute more than 30% of the carbon of the grain [10]; however, some researchers found that the contribution of the flag leaf to grain had been overestimated, with only 3–18% of grain carbon assimilates originating from the flag leaf [11]. These important contributions to grain quality highlight the need to delay senescence and prolong photosynthesis to increase wheat yields.

Different varieties of the same species often differ in how their photosynthetic characteristics change during leaf senescence, indicating that senescence is closely related to genotype. Usually, photosynthetic performance decreases with the decline of chlorophyll^12^, although a slight decrease may not impair photosynthesis [13]. Furthermore, various components of the photosynthetic apparatus may exhibit differences during senescence [8, 13]. Faliang Zeng [14] found that the energetic connectivity of the photosystem II (PSII) units was not as strongly affected as the electron transport chains, which were inhibited during leaf senescence in *japonica* rice (*Oryza sativa*). Viljevac Vuletić and Španić [15] reported that, in early-senescence wheat varieties, the activity of the donor side of PSII and the stability of the PSII units decline earlier than most other photosynthetic indexes. It has been reported that the degradation of individual chloroplast proteins during senescence is mostly uncoordinated and independent of their inherent stability during earlier developmental stages [16]. Moreover, the degradation of chlorophyll-binding proteins lags behind chlorophyll catabolism [16]. Consequently, we assume that different phases of photosynthetic electron transport might decrease at varying rates, partly depending on the speed of chlorophyll degradation.

The prompt chlorophyll a fluorescence (PF) of the leaves increases following illumination, rising from a minimal O point to a maximal P point with two characteristic points (J and I) during the fluorescence rise. The chlorophyll fluorescence induction curve, also known as the OJIP kinetics, is widely used for the nondestructive determination of photosynthesis [17–19]. Photosystem I (PSI) and plastocyanin (PC) can specially absorb 820-nm light in their oxidative states; thus, the oxidative state of PSI and PC can be measured by monitoring the change in modulated 820-nm light reflection (MR) of leaves illuminated with action-spectrum light or far-red light [20–22]. The MR can also be used to investigate the electron transfer between PSI and PSII [20, 23]. Delayed chlorophyll a fluorescence (DF), emitted mainly from PSII, is the result of backward electron transfer in the photosynthetic electron transport chain [24]. DF signals are usually measured using light and dark cycles [25]. The DF intensity decreases in each dark interval, a time-dependent change known as the DF decay curve [22]. The DF signals are measured at the same point in the dark interval to construct the DF induction curve [25]. Since the invention of multifunctional plant efficiency analysis (M-PEA), simultaneously measurement of PF, DF and MR has been popularly used in investigation of the impact of stress factors on the photosynthetic electron transport chain [26, 27].

China is a major wheat-breeding country that releases over 200 new wheat cultivars annually. Previous studies on senescence have typically used fewer wheat varieties, making it challenging to investigate the patterns and reasons for the differential impact on electron transport among various cultivars. Moreover, in the field, senescence is influenced by environmental factors such as temperature [28, 29], nitrogen levels [30, 31], and drought [32, 33], causing it to vary among genotypes with differing stress resistances. In this study, we investigated the flag leaves of 32 wheat cultivars in dark conditions in vitro to simulate senescence in the absence of environmental influence. The simultaneous measurement of PF, DF, and MR was used to study the pattern of variation in photosynthetic electron transport in the flag leaves of winter wheat with different senescence rates and clarify the relationship between the activities of different parts of the photosynthetic electron transport chain in response to chlorophyll content. We aim to deepen the understanding of the dynamics of the photosynthetic electron transport chain during senescence in modern dominant wheat varieties and provide a basis for the future breeding of anti-senescence varieties.

## Materials and methods

### Plant materials and growth conditions

This research was conducted concurrently with a study on genetic differences in photosynthetic electron transport in wheat flag leaves during dark-induced senescence [34]. Thirty-two winter wheat cultivars (*Triticum aestivum*), recently released and popular in the southern Huang-Huai-Hai Plain of China (Table 1), were chosen and planted in the experimental field of Henan Academy of Agricultural Sciences (Yuanyang, Henan, China; 35°00′N, 113°40′E). The experimental field's soil is rich in organic matter and is a slightly alkaline sandy clay. The plot area was 90 m^2^ (30 m long and 3 m wide). On October 10, 2020, seeds were sown at a density of 180 kernels per square meter using a plot planter. The management of the field adhered to the local standard agronomic practices. On the afternoon of May 7, 2021, six flag leaves that were healthy and well-developed, with similar size and shape, were chosen from each variety and taken to the lab for testing. The leaves were placed in a climate-controlled cabinet (total darkness, 25℃) for overnight dark adaptation. The experiment started in the morning of the next day (May 8, 2021). At 8:30 am, the SPAD values, PF, MR, and DF kinetics of the flag leaves were recorded at the start of the experiment, marking the D0 time point. Flag leaves were kept in complete darkness and wrapped with damp cotton towels for 2 and 4 days, after which the same parameters were assessed, marking the D2 and D4 time points.

**Table 1:** Wheat cultivars used in this study

Number	Name	Number	Name
1	Zhongmai 895	17	Zhengmai 0926
2	Zhoumai 36	18	Zhengmai 0943
3	Xinong 979	19	Zhengmai 6694
4	Bainong 207	20	Zhengmai 7698
5	Jimai 22	21	Zhengmai 1354
6	Cunmai 21	22	Zhengmai 1860
7	Wanfeng 269	23	Zhengmai 583
8	Zhengmai 22	24	Zhengmai 20
9	Zhengmai 113	25	Fanmai 8
10	Zhengmai 369	26	Xuke 918
11	Zhengmai 158	27	Zhongmai 578
12	Zhengmai 101	28	Zhoumai 27
13	Zhengmai 27	29	Xinmai 26
14	Zhengmai 379	30	Aikang 58
15	Zhengmai 136	31	Xinong 511
16	Zhengmai 925	32	Bainong 4199

### Measurement of leaf chlorophyll content

Measurements of SPAD values were taken with a SPAD-502 instrument (Minolta Co., Ltd., Japan) at the time points D0, D2, and D4.

### Prompt chlorophyll a fluorescence, delayed chlorophyll a fluorescence and modulated 820 nm reflection measurement

The kinetics of PF, DF, and MR were simultaneously captured using a Multifunctional Plant Efficiency Analyzer (M-PEA, Hansatech, Norfolk, UK), with all leaves dark-adapted for 30 min before the measurements. The actinic light LED emitted wavelengths of 627 ± 10 nm, while the modulated light LED emitted wavelengths of 820 ± 25 nm. The leaves were illuminated for 2 s with a saturating light pulse of 5000 µmol photons m^−2^ s^−1^ intensity emitted by the M-PEA. The device measured PF during the light interval and DF during the dark interval when the actinic light was switched on and off, respectively.

According to the JIP test [23, 35, 36], chlorophyll fluorescence transients were examined using the original data from polyphasic fluorescence transients and the computational formulas provided in Table 2.

**Table 2:** Parameters and formula of rapid chlorophyll fluorescence inducing kinetic curve

Parameter	Method of calculation
*F*_M_	Maximum fluorescence intensity obtained under light after dark adaptation
*F*_O_	Fluorescence intensity at 20 μs of OJIP curve
*F*v	*F*_V_ = *F*_M_* − F*_O_
*F*_*t*_	Fluorescence intensity at *t* time
*F*_K_	Fluorescence intensity at 0.3 ms
*F*_J_	Fluorescence intensity at 3 ms
*F*_I_	Fluorescence intensity at 30 ms
*V*_J_	*V*_J_ = (*F*_J_ − *F*_O_)/(*F*_M_ − *F*_O_)
*V*_I_	*V*_I_ = *(F*_I_ − *F*_O_*)*/(* F*_M_ − *F*_O_)
*M*_O_	*M*_O_ = 4(*F*_300μs_ − *F*_O_)/(*F*_M_ − *F*_O_)
*N*	*N* = *(S*_M_*/S*_S_*)* = *S*_M_ *M*_O_*(1/V*_J_*)*
*Ψ*_O_	*ψ*_O_ = 1 − *V*_J_
*φP*_O_	*φP*_O_ = *F*_V_*/F*_M_ = *(F*_M_* − F*_O_*)/F*_M_
φ*E*_O_	φ*E*_O_ = *ET*_O_/*ABS* = [1 − (*F*_O_/*F*_M_)]∙*ψ*_O_
φ*D*_O_	*φD*_O_ = 1 − *φP*_O_
φ*R*_O_	*φR*_O_ = *φP*_O_ × (1 − *V*_I_)
δR_O_	*δR*_O_ = (1 − *V*_I_)/(1 − *V*_J_)
ABS/RC	ABS/RC = *M*_O_•*(*1/ *V*_J_*)*•(1/*φP*_O_)
RC/CS_m_	RC/CS_m_ = *φP*_O_ × (*V*_J_/*M*_O_) × *F*_M_
PI_ABS_	PI_ABS_ = (RC/ABS) × [*φP*_O_/(1 − *φP*_O_)] × [*ψ*_O_/(1 − *ψ*_O_)]

The MR induction curve of leaves exposed to saturating red light displays a rapid oxidation phase followed by a reduction phase. Maximum decrease in slope (V_ox_, in the range of 1.1–2 ms) and maximum increase in slope (V_RED_, in the range of 30–100 ms) of the MR/MR_0_ was calculated using excel 2019.The oxidation potential of P700, known as Vox, is used to assess the activity of PSI [23, 36].

The points I_1_, I_2_, and D_2_, which are characteristic of the DF induction curve, were evaluated. I_1_ is the first maximum, I_2_ is the second maximum and D_2_ is the lowest point of the curve. The I_2_/I_1_ ratio was obtained from the DF induction curve, while five other DF parameters, L_1_, L_2_, L_3_, τ_1_, and τ_2_, were extracted from the DF decay curve according to method of Gao J (2014).

### Statistical analysis

One-way ANOVA was employed to analyze the effects of dark-induced senescence on SPAD and other parameters (PF, MR, and DF) using IBM SPSS 26.0, followed by a LSD’s multiple range test (α = 0.05).

## Results

### Chlorophyll content in the flag leaves of different wheat cultivars decreased under darkness

**Fig. 1:**
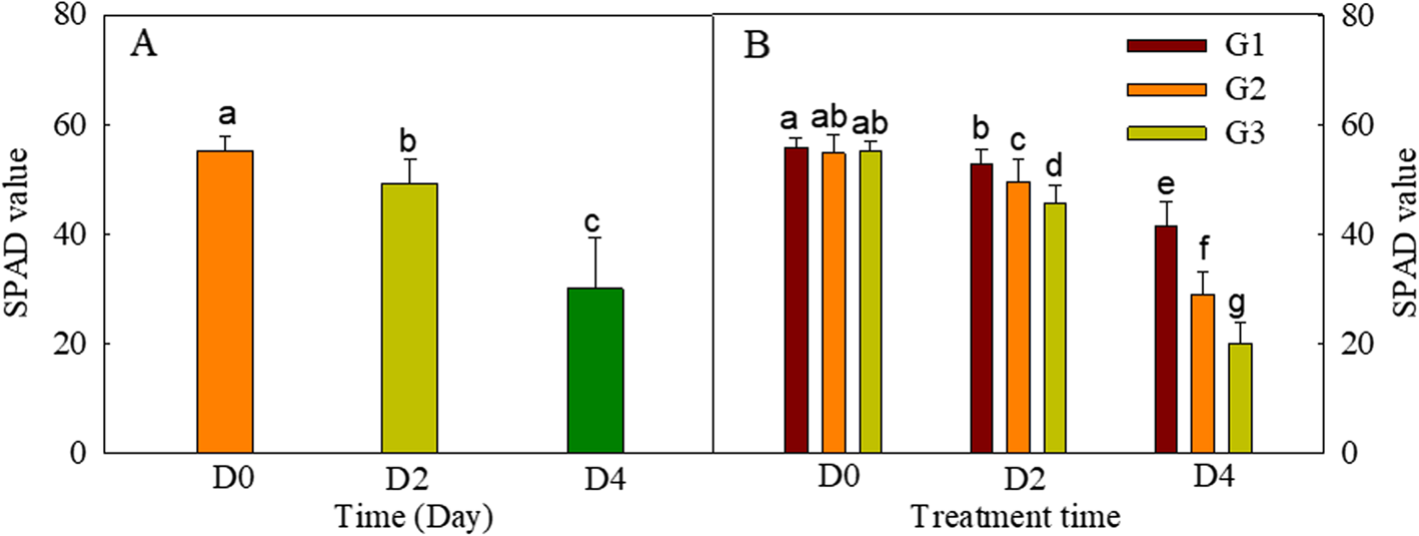
Averaged SPAD values of the flag leaves of wheat cultivars subjected to different durations of darkness. **A** Averaged SPAD values of all wheat cultivars. **B** Average SPAD values of the three groups of cultivars, divided according to their chlorophyll degradation rate during the dark treatment. Different letters indicate significant differences among the leaves of the different groups and treatment periods (*P* < 0.05). The values were presented as means ± SD (A: *n* = 32; B: *n* = 9, 14, and 9 for G1, G2, and G3, respectively)

The parameter SPAD is commonly used to reflect the chlorophyll content in plants [37–39]. We measured the SPAD of the flag leaves of all 32 cultivars at the start of the experiment, and after two and four days of darkness. For all cultivars, the SPAD decreased during the dark treatment (Table 3, Fig. 1A). The 32 cultivars were divided into three groups using the k-means clustering method according to their rate of decrease in SPAD (Table 3). The groups contained 9 (G1), 14 (G2), and 9 (G3) cultivars, respectively (Table 3), with G3 showing the largest rate of chlorophyll decrease and G1 the smallest (Fig. 1B). All three groups started to exhibit significant differences in chlorophyll content on the second day of the dark treatment.

**Table 3:** The SPAD values of wheat cultivars under dark treatment for different time

Cluster results	Cultivar number	The initial SPAD value			The standardized SPAD value			The decrease slop of SPAD
		D0	D2	D4	D0	D2	D4	
1	3	52.65 ± 2.74	43.52 ± 5.37	13.50 ± 5.75	1.00	0.83	0.26	-0.19
1	5	57.20 ± 2.43	53.10 ± 2.30	33.55 ± 11.20	1.00	0.93	0.59	-0.10
1	7	53.17 ± 1.91	48.92 ± 1.62	40.58 ± 5.72	1.00	0.92	0.76	-0.06
1	9	56.80 ± 2.50	46.53 ± 4.54	21.27 ± 5.41	1.00	0.82	0.37	-0.16
1	11	58.20 ± 1.20	57.68 ± 3.39	49.57 ± 6.06	1.00	0.99	0.85	-0.04
1	13	57.78 ± 3.25	50.70 ± 3.05	23.60 ± 5.29	1.00	0.88	0.41	-0.15
1	15	55.25 ± 3.58	53.32 ± 3.07	46.10 ± 5.74	1.00	0.97	0.83	-0.04
1	25	57.38 ± 2.56	50.50 ± 2.57	31.25 ± 10.78	1.00	0.88	0.54	-0.11
1	29	54.52 ± 3.53	50.55 ± 2.73	36.77 ± 6.67	1.00	0.93	0.67	-0.08
2	2	61.45 ± 3.24	53.52 ± 3.64	36.47 ± 10.7	1.00	0.87	0.59	-0.10
2	8	56.17 ± 3.56	51.82 ± 2.49	42.33 ± 8.24	1.00	0.92	0.75	-0.06
2	10	54.88 ± 3.12	44.88 ± 3.50	20.53 ± 5.05	1.00	0.82	0.37	-0.16
2	14	57.93 ± 3.23	52.10 ± 4.05	37.45 ± 10.84	1.00	0.90	0.65	-0.09
2	16	52.58 ± 3.42	46.73 ± 3.04	24.45 ± 6.56	1.00	0.89	0.47	-0.13
2	17	57.60 ± 4.21	56.38 ± 3.78	43.75 ± 5.71	1.00	0.98	0.76	-0.06
2	19	53.46 ± 1.61	52.26 ± 1.47	33.12 ± 15.46	1.00	0.98	0.62	-0.10
2	20	55.85 ± 2.14	49.57 ± 3.22	29.95 ± 6.67	1.00	0.89	0.54	-0.12
2	21	54.77 ± 4.77	46.85 ± 4.88	23.53 ± 9.23	1.00	0.86	0.43	-0.14
2	24	56.22 ± 2.47	52.70 ± 2.70	30.03 ± 9.96	1.00	0.94	0.53	-0.12
2	27	52.52 ± 1.88	51.05 ± 1.77	25.87 ± 7.42	1.00	0.97	0.49	-0.13
2	28	54.72 ± 4.03	49.37 ± 4.79	26.68 ± 10.92	1.00	0.90	0.49	-0.13
2	30	53.43 ± 1.30	48.02 ± 4.36	16.03 ± 7.50	1.00	0.90	0.30	-0.18
2	31	56.12 ± 5.09	47.72 ± 5.49	20.50 ± 8.73	1.00	0.85	0.37	-0.16
3	1	50.33 ± 4.76	45.75 ± 4.88	25.02 ± 6.22	1.00	0.91	0.50	-0.13
3	4	54.97 ± 2.50	52.22 ± 1.99	40.63 ± 9.01	1.00	0.95	0.74	-0.07
3	6	56.68 ± 1.36	42.35 ± 6.96	24.67 ± 11.89	1.00	0.75	0.44	-0.14
3	12	57.12 ± 5.38	47.65 ± 6.94	27.12 ± 13.27	1.00	0.83	0.47	-0.13
3	18	56.43 ± 2.66	54.53 ± 8.03	33.75 ± 12.22	1.00	0.97	0.60	-0.10
3	22	54.83 ± 4.02	52.23 ± 3.03	37.45 ± 4.57	1.00	0.95	0.68	-0.08
3	23	55.52 ± 1.27	49.25 ± 3.94	26.75 ± 2.96	1.00	0.89	0.48	-0.13
3	26	48.18 ± 3.40	37.60 ± 6.41	22.53 ± 6.24	1.00	0.78	0.47	-0.13
3	32	52.68 ± 2.74	39.73 ± 6.62	16.60 ± 3.43	1.00	0.75	0.32	-0.17

Note: Each SPAD value is the average of 6 leaves

Values shown are means ± SD

### PF (OJIP) transient analysis

**Fig. 2:**
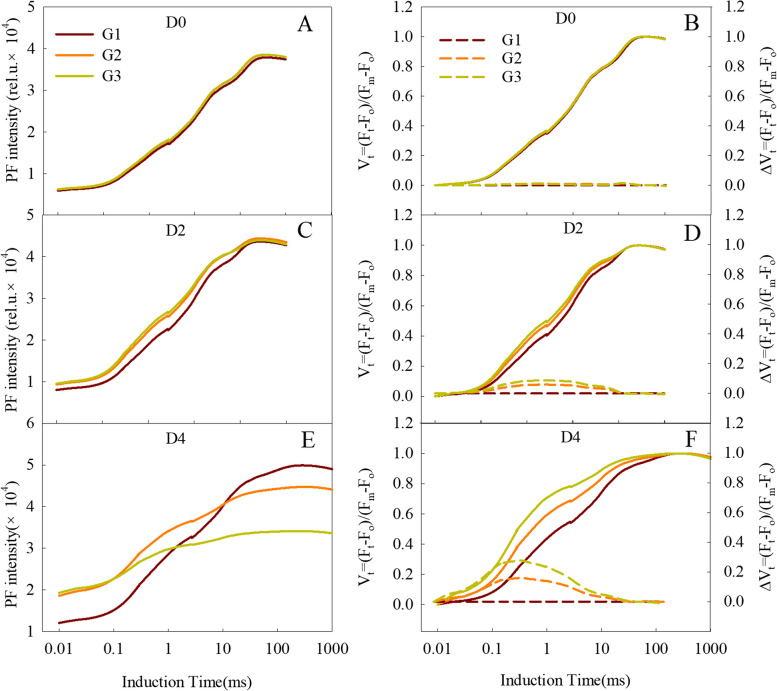
The prompt chlorophyll a fluorescence (PF) transient of the flag leaves of the three wheat cultivar groups. D0, D2, and D4 mean the start, 2nd day, and 4th day of the dark treatment, respectively. (**A**, **C**, **E**): Absolute values of the three wheat cultivar groups. (**B**, **D**, **F**): The solid lines are OJIP curves normalized according to the O–P point, expressed as V_t_ = (F_t_ − F_O_)/(F_M_ − F_O_). The dashed lines are OJIP curves plotted as difference kinetics, expressed as ΔVt = Vt ^G2 or G3^–Vt.^G1^. Every curve is the average of the curves of each wheat cultivar in that group (G1, G2, or G3)

The PF transients were averaged for each of the three wheat groups, and all curves exhibited the expected points O, J, I, and P, presenting a typical OJIP transient (Fig. 2A,C,E). The O points of all three wheat groups increased with the duration of the dark treatment. The average O points in the leaves of the G2 and G3 cultivars treated for two and four days were significantly higher than that of G1. The maximum fluorescence intensity (F_M_) in the G1, G2, and G3 leaves showed no significant change within two days of dark treatment, while after a four-day treatment the Fm values were largest in G1, followed by G2, with the smallest observed in G3 (Fig. 2A,C,E). When the OJIP transient was normalized by the O and P points, the J and I points were higher in the leaves of the G2 and G3 cultivars than in the G1 leaves. Changes in the J point represent the electron transfer from Q_A_^–^ to Q_B_ [24, 40], while the I point represents electron transfer between PQ and PSI [41, 42]. Both the J and I points are highly sensitive to various abiotic stresses, including drought [43], salt [44], and high temperature [45]. The increases we observed in the J and I points of the senescence flag leaves reflected an inhibition of the electron transfer from Q_A_^–^ to Q_B_ and PQ to PSI. The differences in the J and I points among the three groups were amplified with the delaying of the treating time, and were more pronounced when the OJIP curves were again normalized (Fig. 2B,D,F), suggesting that the inhibition of electron transfer from Q_A_ to Q_B_ and PQ to PSI are distinguishing characteristics of senescence process in the flag leaves.

**Fig. 3:**
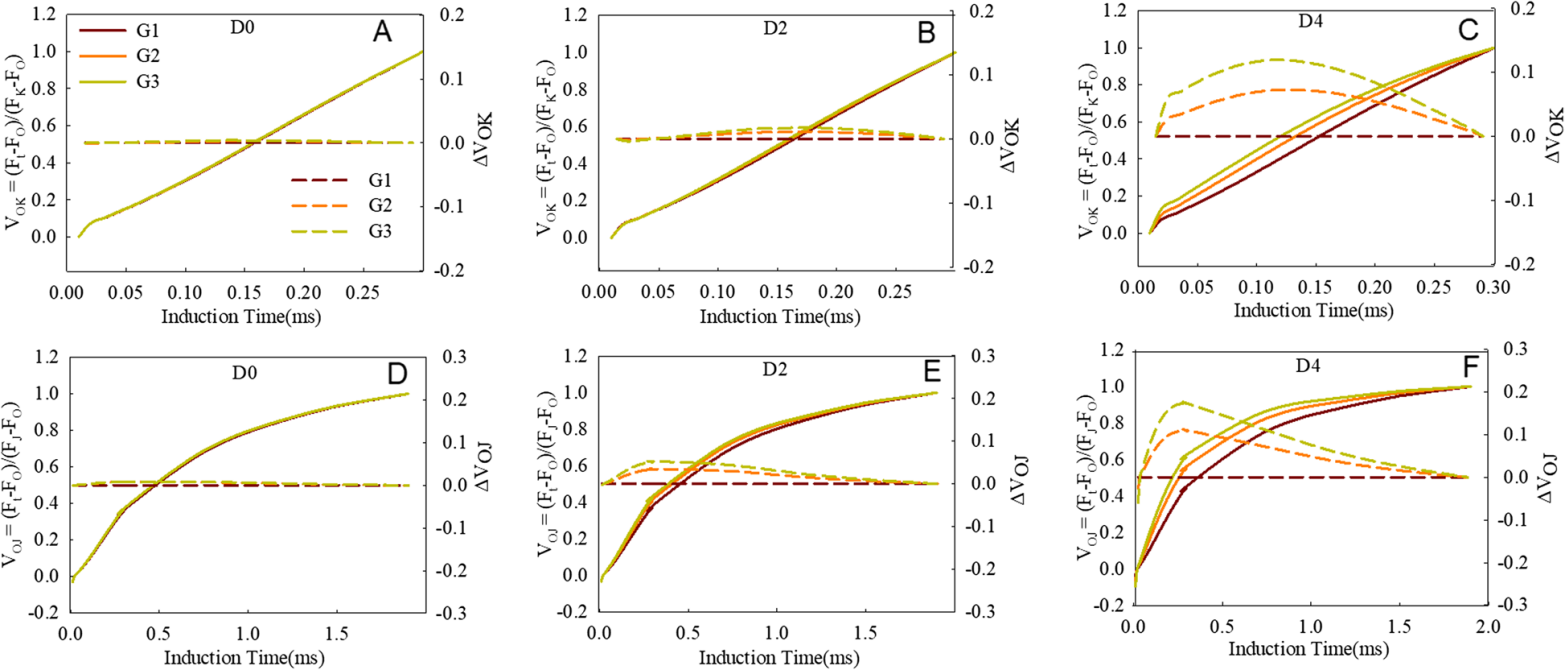
The shape of the OK and OJ bands of the flag leaves of the three wheat groups during the dark treatment. The solid lines are OJIP curves normalized according to the O–K and O–J points, expressed as V_OK_ = (F_t_ – F_O_)/(F_K_ − F_O_) and V_OJ_ = (F_t_ – F_O_)/(F_J_ − F_O_), respectively. The dashed lines are OJIP curves plotted as difference kinetics, expressed as ΔVt = Vt ^G3 or G5^ – Vt ^G1^. Every curve is the average of the curves of each wheat cultivar in that group (G1, G2, and G3). D0, D2, and D4 mean leaves treated for 0, 2, and 4 days, respectively

We extended our investigation by normalizing the PF in the O–K, and O–J phases (Fig. 3). As shown in Fig. 3A–C, the fluorescence data were double-normalized by F_O_ (20 µs) and F_K_ (300 µs). An additional step around 150 µs, known as the L-band, can be observed through this subtraction. The L-band indicates the coupling of PSII units and the energy exchange between them [24, 46]. When the fluorescence data were double normalized by F_o_ (20 µs) and F_J_ (2 ms), a K-band could be observed (Fig. 3D–F). The K-band represents the activity of the PSII donor side [47]. The L-band and K-band showed similar trends, both were greater with longer dark treatments, and rose greater in the wheat lines with faster senescence (Fig. 3), indicating that the stability of the PSII donor side and the association of the PSII units are closely related to the rate of senescence in the wheat cultivars.

**Fig. 4:**
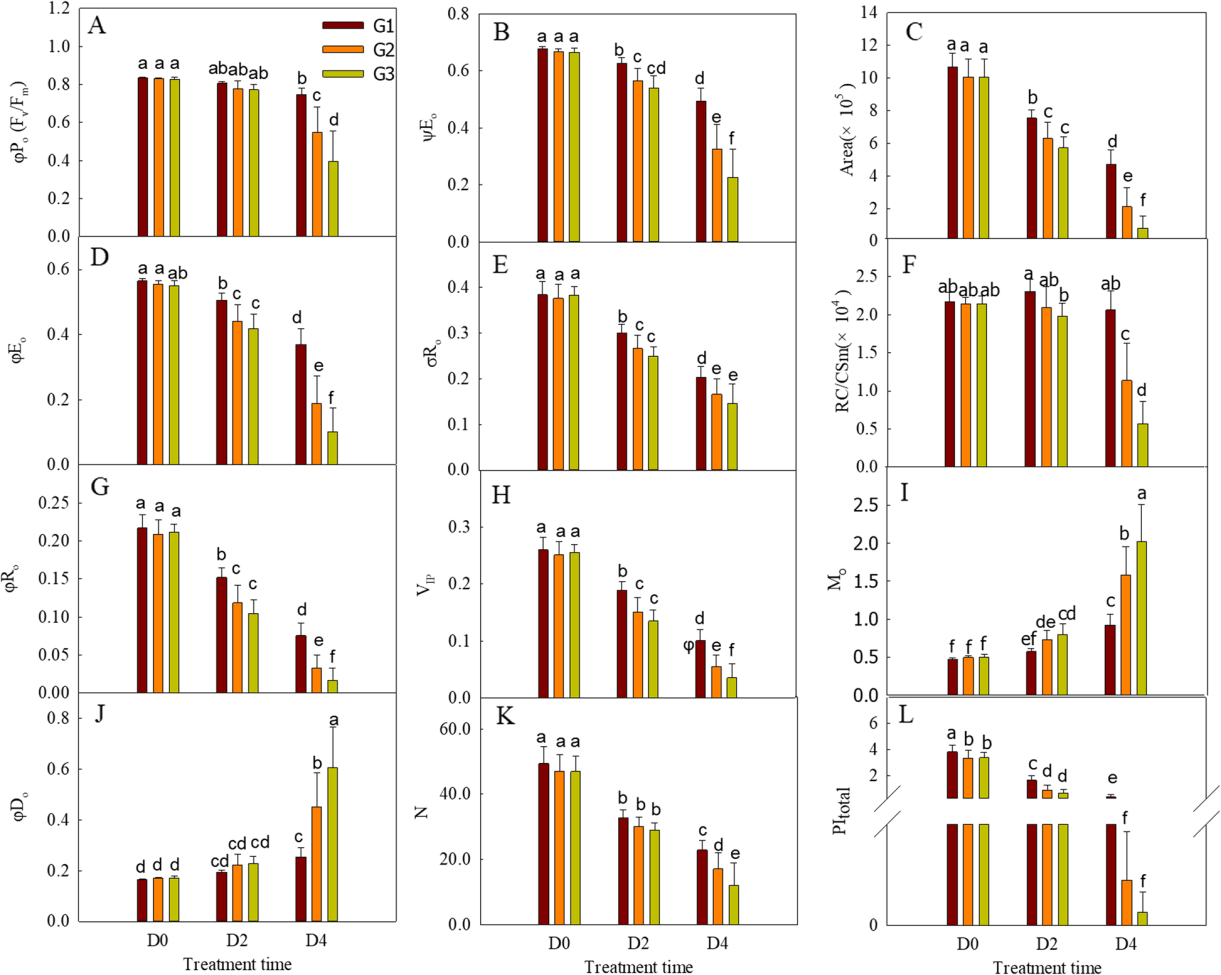
Parameters derived from the prompt chlorophyll a fluorescence (PF) transients of the three wheat cultivar groups under dark treatment for different durations. Different letters above the bars indicate significant differences among the groups and treatments at the *P* < 0.05 level. D0, D2, and D4 mean leaves treated for 0, two, and four days, respectively. The values were presented as means ± SD (*n* = 9, 14, and 9 for G1, G2, and G3, respectively)

In order to quantitatively analyze the different responses of the photosynthetic transport chains of the wheat cultivars with varying senescence rates during the dark treatment, several parameters were obtained from the OJIP transient using the JIP test (Table 2). The three wheat groups showed different sensitivities to the dark treatment. The parameters φPo, φEo, φRo, PI_total_, V_IP_, ψEo, δRo, Area, N, and RC/CS_m_ decreased with the duration of the darkness, while the parameters φDo and M_O_ both gradually increased when the darkness was prolonged (Fig. 4). The G3 group showed the greatest changes under the dark treatment, followed by G2 and finally by G1 (Fig. 4).

### MR/MRo transient analysis

**Fig. 5:**
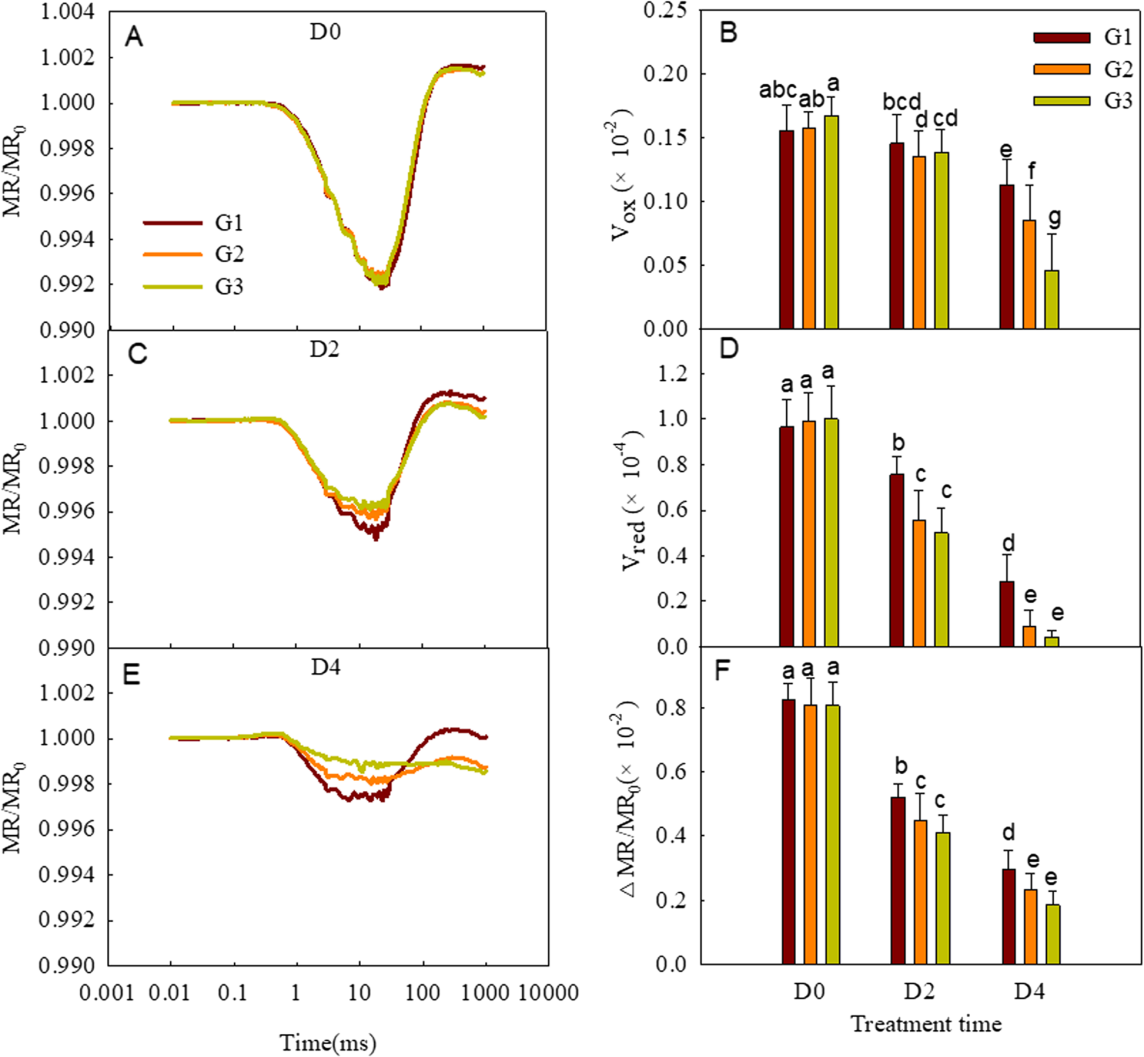
The modulated 820-nm reflection kinetics (MR) (**A**, **B**, **C**) and related parameters (**D**, **E**, **F**) of the different wheat groups (G1, G2, and G3) under the dark treatment for different periods of time. The MR signals were plotted on a logarithmic time scale. Every curve is the average of the curves of each wheat cultivar in the group (G1, G2, and G3). Different letters above the bars indicate significant differences among different groups and different periods of the dark treatment at the *P* < 0.05 level. D0, D2, and D4 mean leaves treated for 0, 2, and 4 days, respectively. **A**, **C** and **E**: Each curve is the averaged curves of the cultivars of the corresponding group. **B**, **D** and **F**: Each bar is presented as means ± SD (*n* = 9, 14, and 9 for G1, G2, and G3, respectively)

As oxidized PSI and PC specifically absorb 820-nm light wavelengths, the redox state of these photosynthetic components can be dynamically monitored as changes in the MR of the leaves [48, 49]. Therefore, P700 and PC are in their reduced states after fully adapting to darkness and gradually oxidize upon illumination. Subsequently, PSI and PC are rapidly reduced by electrons coming from PSII; therefore, MR kinetics consist of an initial decline phase and then an ascending phase. The reduction rates of PC and PSI are equal to their oxidation rates at the lowest point of the MR kinetics. In the present study, we measured the MR kinetics of the different wheat groups, and the relative parameters V_OX_, V_RED_, and MR/MR_0_ were derived (Fig. 5). The rates of decline and increase of the MR both increased with the duration of the dark treatment (Fig. 5), with the G3 wheat showing the greatest MR sensitivity to the dark treatment, followed by G2, with G1 being the least sensitive.

### DF induction and decay transient analysis

DF occurs due to the backward flow of electrons reaching the PSII reaction centers, leading to charge recombination and the re-excitation of PSII antenna chlorophyll [22, 25, 50]. Using the M-PEA, DF was measured concurrently with PF and MR in this study. The measurement was performed using an alternate light/dark cycle, where PF and MR were recorded in the light periods and DF was recorded in the dark intervals. The DF signal showed a polyphasic decrease in each dark interval, which forms the DF decay curve (Figure S1).

**Fig. 6:**
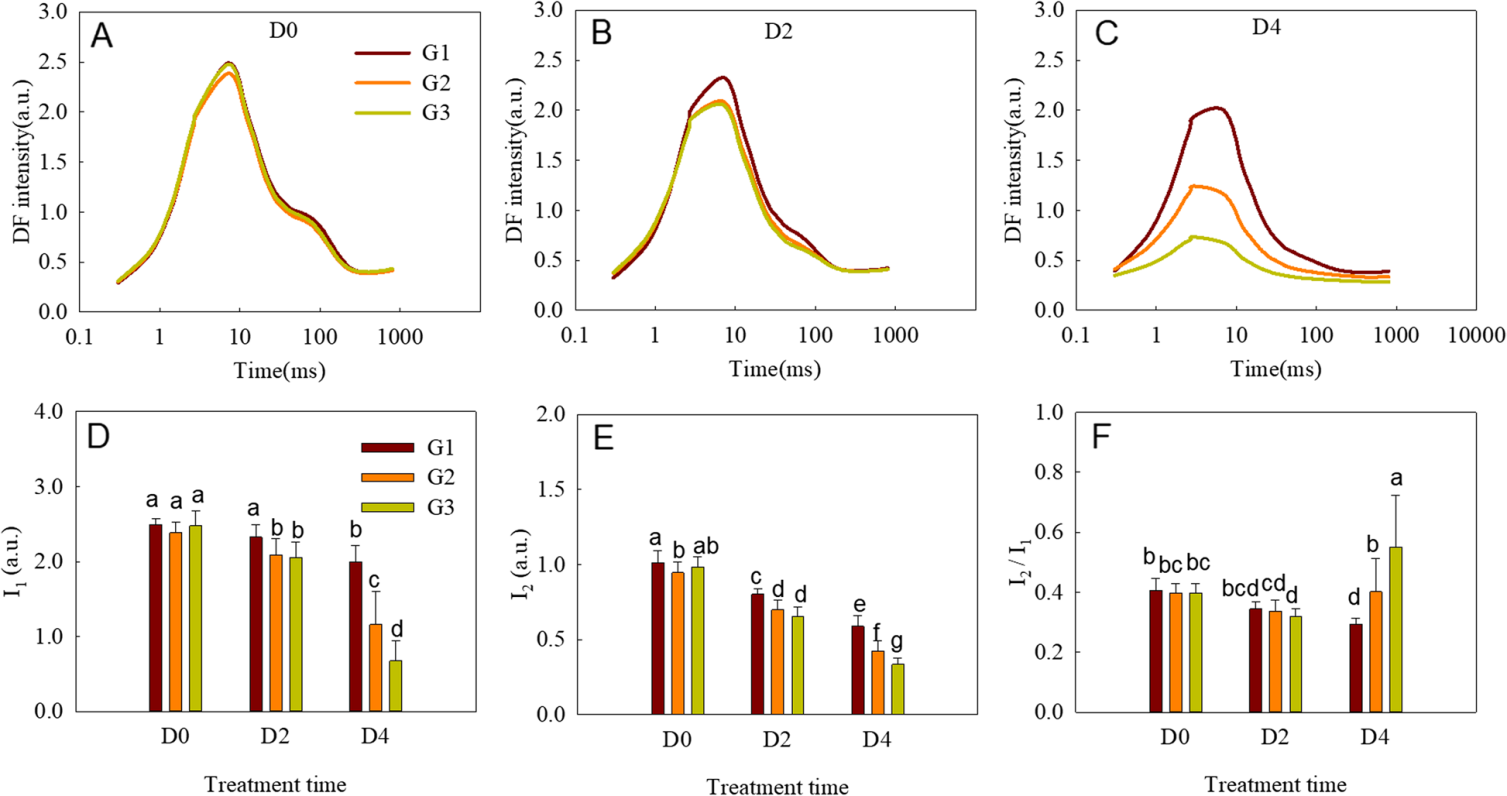
Delayed chlorophyll a fluorescence (DF) of the three wheat cultivar groups subjected to different periods of darkness. **A**–**C** DF induction kinetics at 20 µs. **D**–**F** I_1_, I_2_, I_2_/I_1_ derived from the DF induction kinetics at 20 µs. Every curve is the average of the curves of each cultivar of the wheat group (G1, G2, and G3). D0, D2, and D4 mean leaves treated for 0, 2, and 4 days, respectively. Each curve was the average curves of the cultivars of the corresponding group. Each bar is presented as means ± SD (*n* = 9, 14, and 9 for G1, G2, and G3, respectively). Different letters above the bars indicate significant differences among the different groups and treatments at the *P* < 0.05 level

In the present study, the signal measured after a 20-μs delay from the start of each dark interval was used to construct the DF induction curves. The DF induction curves showed an increase from an initial minimum (Do) to a maximum (I_1_) at about 7 ms, then decreased to a second maximum (I_2_) at about 100 ms and finally reached a plateau (D2) (Fig. 6 A-C). It has been suggested that I_1_ represents the accumulation of a relatively large proportion of PSII reaction centers in the S_3_Z^+^P_680_Q_A_^−^ state, while I_2_ reflects the reopening of the PSII RCs by the electron transfer from reduced Q_B_ to PQ [25, 51]. When a longer dark treatment was used, the increased DF from D_0_ to I_1_ and decreased rate of I_1_ to D_2_ on the induction curve were both decreased relative to shorter dark treatments, resulting in lower I_1_ and I_2_ values. The parameters I_2_/I_1_ of the leaves slightly decreased after a two-day treatment in the dark, but increased when the darkness treatment lasted four days. The extent of the I_2_/I_1_ increase of the three wheat groups on the 4th day was greatest in G3, then G2, and smallest in G1 (Fig. 6 D-F).

Five DF parameters, namely L_1_, L_2_, L_3_, τ_1_, and τ_2_, were extracted from the I_1_ of the DF decay curves (Table 4; Figure S2). L_1_, L_2_, and L_3_ signify the amplitudes of three emission components, whereas τ1 and τ2 signify the lifetimes of the first two emission components 23, 26. In the present study, the three wheat groups subjected to darkness for different duration had similar τ_1_ and τ_2_ values. The τ1 value was about 20 µs, corresponding to the PSII reaction centers in the ZP_680_^+^Q_A_^−^ state [25]. The τ_2_ value was about 300 µs, corresponding to the PSII reaction centers in the Z^+^P_680_Q_A_^−^Q_B_ state [25]. The leaves of all three wheat groups showed decreasing L_1_ and L_2_ values during the dark treatment, with the G3 leaves showing greater decreases than those of G2 and G1; L_1_ and L_2_ of G1 were the most insensitive to the dark treatment. The above results suggest that dark-induced senescence decreased the ZP_680_^+^Q_A_ and Z^+^P_680_Q_A_^−^Q_B_ states in the PSII reaction centers, but that this reduction is reduced in stay-green wheat cultivars in comparison with cultivars that undergo earlier senescence.

**Table 4:** Parameters for DF decay curves were obtained by fitting the experimental data to the time function DF (t) = L_1_ × exp (-t/τ_1_) + L_2_ × exp (-t/τ_2_) + L_3_, with L_1_, L_2_ and L_3_ being the amplitudes (in relative units) of the kinetic components

Duration of dark treatment	Classification of wheat varieties	Parameters				
		L_1_	L_2_	L_3_	τ_1_	τ_2_
D0	G1	30,087.26 ± 987.41a	6836.78 ± 463.16a	1696.2 ± 106.03a	0.02 ± 0.001	0.304 ± 0.006
	G2	28,459.8 ± 2025.48a	6770.55 ± 432.38a	1627.87 ± 129.72a	0.02 ± 0	0.31 ± 0.02
	G3	28,951.63 ± 1669.85a	7079.7 ± 728.46a	1683.29 ± 61.38a	0.02 ± 0	0.3 ± 0.01
D2	G1	27,603.04 ± 2099.93ab	6510.82 ± 773.52a	1742.19 ± 155.79a	0.02 ± 0	0.31 ± 0.01
	G2	25,197.78 ± 2633.8bc	5492 ± 998.63b	1689.21 ± 126.26a	0.02 ± 0	0.3 ± 0.02
	G3	24,440.33 ± 2038.82 cd	5476.66 ± 1108.01b	1743.18 ± 178.13a	0.02 ± 0	0.31 ± 0.02
D4	G1	21,724.94 ± 3213.56d	5255.85 ± 727.19b	1781.76 ± 160.54a	0.02 ± 0	0.33 ± 0.02
	G2	12,227.11 ± 5043.11e	2589.99 ± 1299.67c	1179.84 ± 393.96b	0.02 ± 0	0.27 ± 0.04
	G3	6611.06 ± 3160f	1359.2 ± 724.97d	709.36 ± 252.21c	0.02 ± 0	0.21 ± 0.07

Note: The values were presented as means ± SD (A: *n* = 32; B: *n* = 9, 14 and 9 for G1, G2 and G3, respectively)

Different letters (a, b, c) indicate significant differences between different cultivar groups and treating time at the 0.05 level

τ1 and τ 2 are lifetimes (in ms) of L_1_ and L_2_, respectively. Each value is the average of each wheat group

### Different sensitivity of electron transport chain to dark induced senescence

**Fig. 7:**
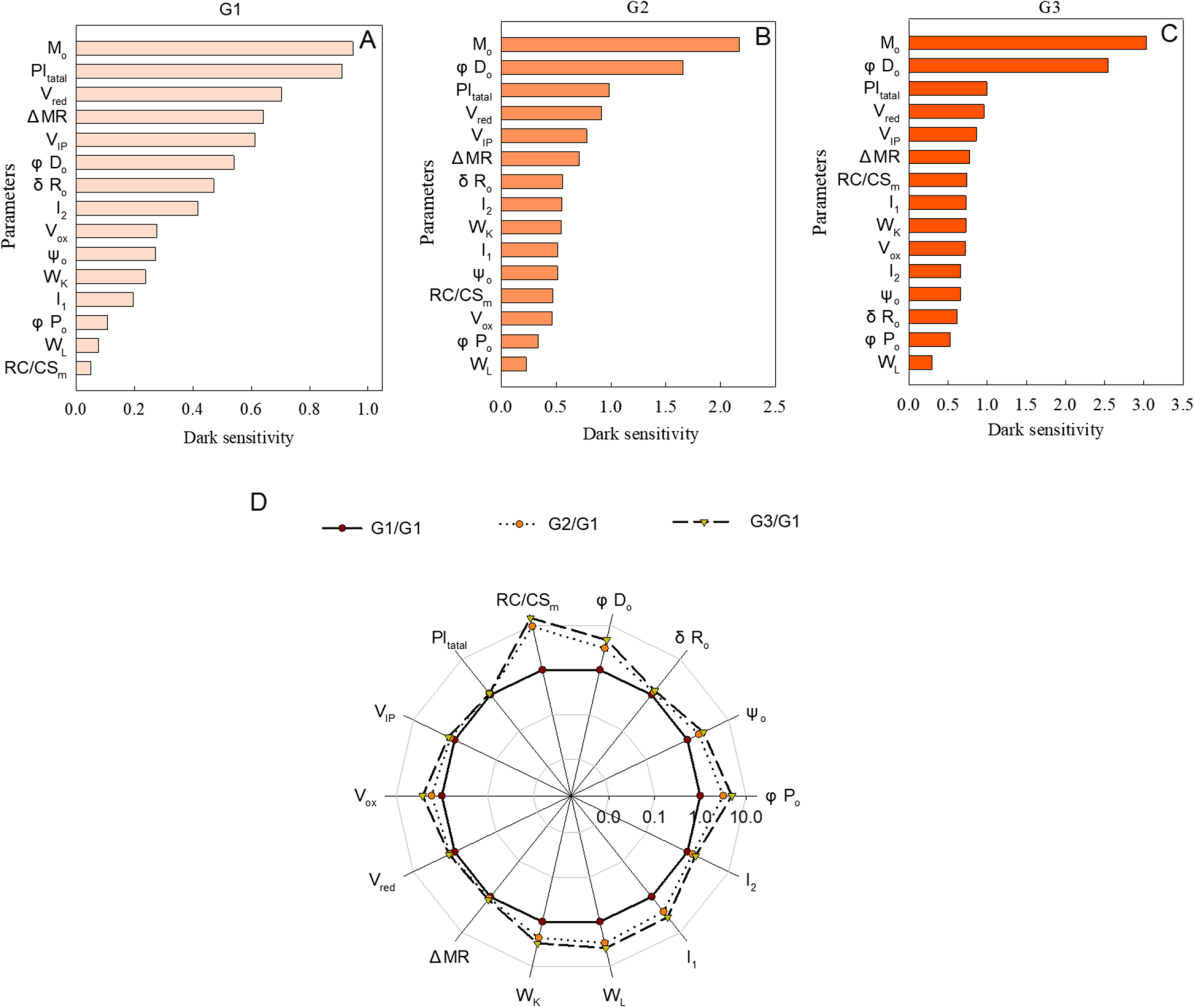
The sensitivity of different parameters of different cultivar groups to dark induced senescence. **A**: parameters of G1 wheat group; **B**: parameters of G2 wheat group; **C**: parameters of G3 wheat group. **D**: Standardization of the dark-induced changes of different parameters of the three cultivar groups. Parameter of the flag leaves measured on D0 and D4 were used to calculate dark-induced changes, as (D4-D0)/D0. In figure **A**-**C**, the parameters were ordered according to the size of the corresponding value. In figure **D**, the dark-induced changes of different parameters were normalized according to G1

To investigate the effect of chlorophyll degradation rate on the sensitivity of different phase of photosynthetic electron transport chain to senescence under dark treatment, the changes of chlorophyll fluorescence parameters of different wheat populations (G1, G2 and G3) were calculated. The results showed that the sensitivity of parameters presented energy absorption and transport (φPo, ψEo and σRo) were enlarged in order for G1 (Fig. 7 and Table S1). In G2 and G3, φPo and ψEo increased faster than σRo, and the sensitivity of ψEo greater than that of σRo in G3 wheat groups (Fig. 7 and Table S1). The appearance of I_2_ is closely linked to the function of PSII to transmit electrons, while I_1_ is mainly influenced by PSII. Compared to G1, the increasing extent of dark induced changes of I_1_ in G2 and G3 were larger than that of I_2_, and in G3, the dark induced changes of I_1_ were larger than I_2_. When the dark induced changes of the parameters in G2 and G3 were normalized to G1, we found the difference of PSI related parameters (σRo, V_IP_, I_2_, ΔMR) among G1, G2 and G3 were lesser than PSII related parameters (φPo, RC/CSm, φDo, W_L_, I_1_), which suggest the sensitivity of PSI and PSII to dark induced senescence were greatly affected by chlorophyll degradation rate (Fig. 7 D and Table S2).

## Discussion

### Dark-induced senescence

Darkness can induce senescence-like physiological processes in plants, a phenomenon which has been widely used in the study of maize (*Zea mays*) [13], *Arabidopsis thaliana *[52], and tomato (*Solanum lycopersicum*) [53]. The degradation of chlorophyll is generally considered an important marker of plant senescence. In this study, the chlorophyll levels (SPAD value) in the flag leaves of the 32 cultivars showed varying degrees of degradation ratio between –0.1859 and –0.04 (Table 3), indicating that senescence was induced in all cultivars. Chlorophyll degradation and the decline of photosynthetic ability are not always synchronous in the senescence process of leaves; sometimes, cultivars with higher chlorophyll contents may have lower photosynthetic abilities during senescence [13]. Classifying multiple varieties according to their senescence rate to study the photosynthetic characteristics of wheat cultivars can remove individual effects, providing insight into the characteristics of photosynthetic function during senescence in modern wheat cultivars. In the present study, the largest proportion (43.75%) of the 32 varieties had a moderate senescence rate, while the fastest and slowest senescence types were observed in nine varieties (28.125%) each (Table 1), which suggest most of the bred varieties are medium anti-aging varieties in south of the Huang-Huai-Hai Plain of China.

### Changes of photosynthetic electron transport during senscence

The OJIP transient is often used to study photosynthesis under environmental stresses, such as drought [50, 54], high temperature [21], low temperature, and salt stress[ 18, 55]. In the present study, the shapes of the OJIP transients of the different wheat cultivar groups displayed typical changes under darkness, with the basic steps of O-J-I-P (Fig. 2A,C,E). F_O_ represents the fluorescence emission from the PSII antenna chlorophyll molecules and the decay before the excitons reach the reaction centers [56]; thus, the increased O point always indicates a physical separation of PSII from the associated pigments. In this study, the Fo of the leaves in the rapidly senescing cultivars increased more than in cultivars with lower senescence rates during the dark treatment (Fig. 2), which suggests that the associated pigment antennae separated from the PSII reaction centers during senescence and to a much greater extent in the fast-senescence cultivars. F_m_ (F_P_) is the point in the OJIP transient at which the PSII reaction centers have been fully closed under saturating light. The decrease of F_P_ under dark treatment for 2 and 4 days might be caused by the reduced availability of active PSII reaction centers, and/or the denaturation and degradation of chlorophyll proteins [57, 58]. RC/CS_m_ indicates the active RCs per cross-section [22, 59]. As RC/CSm and SPAD in the flag leaves both decreased during senescence (Fig. 4), the decrease of F_m_ in this study might be caused by the complex effects of the reduction of active PSII reaction centers and the degradation of chlorophyll proteins. The inactive PSII reaction centers would act as excitation traps to dissipate excitation energy [60]. Consequently, the increased quantum yield of energy dissipation (φDo) in the flag leaves during senescence further supports the decrease in active PSII reaction centers (Fig. 4). The above results suggest that both the PSII reaction centers and antennae chlorophyll molecules were damaged during the senescence of the flag leaves, and that the extent of the damage is closely related to the speed of senescence.

ψEo reflects the probability that a trapped exciton moves an electron into the electron transport chain beyond Q_A_^–^. δR_O_ reflects the probability that an electron transferred from the intersystem electron carriers to reduce end electron acceptors at the PSI acceptor side [21]. The values of ψEo and δRo decreased significantly as senescence progressed, which was amplified in rapidly senescing cultivars (Fig. 4), consistent with the changes of the J and I points (Fig. 2). Inhibiting electron transport from Q_A_^–^ to Q_B_ and PQ to PSI could result in a lower quantum yield for electron transport (φEo) and a decrease in the reduction of end electron acceptors at the PSI acceptor side (φRo). Decrease of PQ pool on PSII acceptor side (Area), accelerating accumulation of Q_A_^–^ (M_0_) and decrease of Q_A_^–^ turnover (N) further indicate the inhibition of electron transport from Q_A_^–^ to its downstream electron carriers during dark-induced senescence. The activity and energy grouping extent on the donor side are key factors influencing the capacity of PSII to transfer electrons downstream [50]. In this study, both the activity of the oxygen-evolving complex and the energy exchange between PSII units diminished during dark-induced senescence (Fig. 3), which indicate that the activity of the oxygen-evolving complex and the energy exchange between PSII units partly contributed to the decline in PSII capacity for electron transport.

The change in the slope of the MR during the decreasing phase (V_OX_) has been reported to relate to the PSI activity and is probably affected by variation in the size of the PSI antennae [61]. In the present study, V_OX_ decreased in the flag leaves during the senescence induced by the dark treatment, with a greater decrease observed in the wheat cultivars undergoing earlier senescence (Fig. 5). Our findings suggest that the PSI activity of the flag leaves decreased during senescence, which is more pronounced in the rapidly senescing cultivars, consistent with the changes of the JIP parameters δRo and V_IP_. Following the rapid-decrease phase, the MR slowly rose to a relatively stable state. The maximum rising rate of the MR (V_RED_) reflected the capacity of PSII to pump electrons to PSI [62]. Similar to the changes in V_OX_, V_RED_ decreased during dark-induced senescence, which suggested the activity of the PSI donor side was decreased, consistent with the decreased activity of PSII and its acceptor side (φPo, ψEo, and M_0_). MR/MR_0_ is the result of the balance of the redox and reduction of PSI and PC. The decrease of the ΔMR/MR_0_ during senescence suggests that PSI is more damaged than PSII, consistent with the change of φPo and δRo (Fig. 6)

As a supplemental technique to PF and MR, DF is frequently used to study changes in the photosynthetic electron transport chain of plants during different development stages or under varying environmental stresses.The microsecond-amplitude (20 µs) DF is closely related to the concentration of the ZP_680_^+^PheoQ_A_^–^ state [63, 64], which depends on the amount of P680 and the activity of both the PSII donor side and the acceptor side. The maxima I_1_ is parallel to the decreasing phase of the MR curve and the I–P phase of the PF transient. The appearance of I_1_ can be related to two phenomena: one is the accumulation of certain light-emitting states of the PSII reaction center, and the other is the increased electrical gradient formed by PSI when P700 is oxidized [65, 66]. Three DF components, L_1_, L_2_, and L_3_, were obtained by the deconvolution of DF decay curves at I_1_. L_1_ and L_2_, predominant components of I_1_, represent the amount of the ZP_680_^+^Q_A_^−^ and Z^+^P_680_Q_A_^−^Q_B_ states, respectively [63, 64]. L_1_ is usually related to the electron transport from Z to P_680_^+^ on the PSII donor side, and L_2_ is mainly affected by the electron transfer from Q_A_ to Q_B_ on the PSII acceptor side [23, 65]. In the present study, both L_1_ and L_2_ decreased during dark-induced senescence, suggesting that the PSII donor and acceptor sides were both damaged, which is also supported by the results of the PF and MR analyses. Moreover, the decrease of active PSII reaction centers (RC/CS_m_) and increase of the electrical gradient may also have contributed to the decrease of I_1_ during the senescence of the flag leaves. I_2_, parallel to the I–P phase of the OJIP transient, is related to the accumulation of the Z^+^P_680_Q_A_^−^Q_B_ state during the PQ pool reduction [63, 64]; thus, I_2_ is closely related to the activity of both PSI and the electron transfer at the PSII acceptor side [25]. I_2_ decreased during senescence, indicating that both the PSI donor side and electron transfer at the PSII acceptor side were inhibited, which is consistent with the results of the PF and MR analyses. Studies have shown that the DF parameter I_2_/I_1_ is linked to the restriction of electron donation on the donor side of PSII [50, 64, 67]. In the present study, I_2_/I_1_ increased during dark-induced senescence, which further indicates that the donor side of PSII was damaged, consistent with the PF and MR results.

Collectively, the above PF, MR and DF parameters demonstrates that dark induced senescence decreased the number of active PSII RCs, damaged both the donor and acceptor sides of PSII, impaired the connectivity between independent PSII units, limited electron transport beyond Q_A_, and destroyed the OEC in wheat flag leaves. These effects, in combination, lead to decreased of activity of entire electron-transport chain, as reflected in the decrease in PI_total_ (Fig. 4L). The results from the three signals of OJIP, MR, and DF in photosystems corroborate each other and provide much more detailed information on changes in the photosynthetic electron chain during senescence than any individual signal alone, as reported in previous studies on other crop species and environmental stresses [50, 68].

### Different sensitivity of electron transport chain to dark induced senescence

φPo, φEo, and φRo represent the maximum quantum yield of the primary photochemistry, the quantum yield of electron transport, and the quantum yield for reduction of the end electron acceptors at the PSI acceptor side (at t = 0), respectively [22]. In the present study, regardless of the variety type, the sensitivity of the three parameters to the decreased chlorophyll content during senescence can be ordered as follows: φPo < φEo < φRo (Fig. 4A,D and G). This suggests that the sensitivity of the photosynthetic electron transport to senescence gradually increased from the upstream to the downstream components. In spite of this, the sensitivity of these photosynthetic processes varied in cultivars with different chlorophyll degradation rate (Fig. 7 A-C; Table S1). Other parameters like RC/CS_m_ and W_K_, I_1_ and I_2_ also appeared the similar phenomenon, the order of which changed when the chlorophyll degradation rate is altered. These results might suggest that chlorophyll degradation rate is a major determinant of the coordination at the different stages of the photosynthetic electron transport in senescence of wheat leaves. During the aging process of leaves, chloroplasts disintegrate and photosynthetic enzymes and proteins degrade, and the degradation rates of the various components of the photosynthetic electron transport chain are not uniform [16, 69]. The diverse responses of different parameters in the present study could reflect the differing degradation rates of the protein components of the electron transport chain. Moreover, the change of activity of different phase of electron transport is main reason for photoinhibition and ROS generation in chloroplast. For example, in the presence of both low temperature and low light, the PSI in cold sensitive plants is more prone to photoinhibition because of excessive electron transported to PSI due to less inhibition of PSII [70]. In high temperature, oxygen-evolving complex is more susceptible to injury, which always results in greater photoinhibition of PSII [71, 72]. In the present studies, the sensitivity of PSI and PSII to dark induced senescence were greatly affected by chlorophyll degradation rate. Thus, we hypothesize that the differential responses of various photosynthetic electron transport processes to rates of chlorophyll degradation could be a key factor contributing to variations in photoinhibition among wheat cultivars, especially during the senescence phase. If this is the case, senescence traits should be considered in future breeding for stress tolerance. However, this hypothesis requires confirmation through further field research in the future.

## Conclusions

**Fig. 8:**
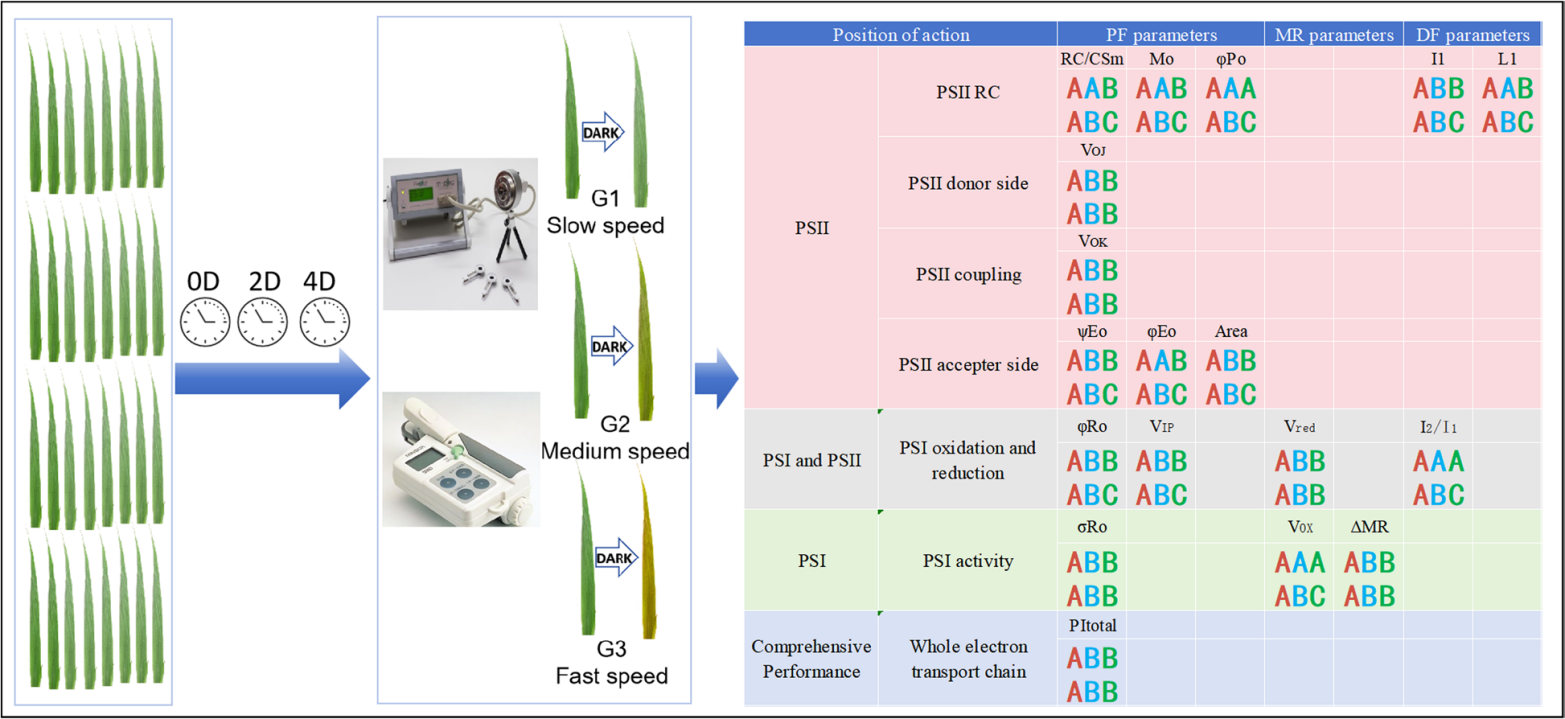
A schematic diagram of the experimental design and results. The letters (**A**, **B**, **C**) signify distinctions among various cultivar groups. The initial and subsequent lines of letters within each cell (with parameter) depict the results of 2 days and 4 days, respectively. Red, blue, and green letters symbolize G1, G2, and G3 cultivar groups, respectively

In conclusion, the PF, DF, and MR characteristics were simultaneously measured and analyzed, revealing that the PSII donor side, PSII unit coupling, PSII reaction center, PSII acceptor side, and PSI were all damaged during dark-induced senescence. The sensitivity of photosynthetic electron transport to senescence gradually increased from the upstream to downstream electron carriers at the PSII acceptor side. The extent of the decrease in the activities of different parts of the photosynthetic electron transport chain during senescence was dependent on the chlorophyll degradation rate of the wheat cultivars (Fig. 8). The three independent signals provided strong mutual support for each other. Additionally, we speculated that differences in the response of different processes of photosynthetic electron transport to chlorophyll degradation rates might be an important factor influencing the differences in photoinhibition under stress among wheat cultivars especially in senescence process.

## Supplementary Information

Supplementary Material 1.

Supplementary Material 2.

## Data Availability

No datasets were generated or analysed during the current study.
