# Supplementary material for: Differential Sensitivity of Photosynthetic Electron Transport to Dark-Induced Senescence in Wheat Flag Leaves
Source: BMC Plant Biol. 2025 May 16;25:650. doi: 10.1186/s12870-025-06624-5 (PMC12082866; doi:10.1186/s12870-025-06624-5)
Supplement: Supplementary file 1 — Supplementary Material 1. [file 12870_2025_6624_MOESM1_ESM.docx]

**Table S1. Sensitivity of different parameters of Tree wheat groups to darkness.**

| Sequence | G1 | | G2 | | G3 | |
| --- | --- | --- | --- | --- | --- | --- |
|  | Parameter | Sensitivity | Parameter | Sensitivity | Parameter | Sensitivity |
| 1 | Mo | -0.95 | PItatal | 0.98 | PItatal | 1.00 |
| 2 | PI_tatal_ | 0.91 | Mo | -2.17 | Mo | -3.03 |
| 3 | V_RED_ | 0.70 | φDo | 1.66 | φDo | -2.54 |
| 4 | ΔMR | 0.64 | V_RED_ | 0.91 | V_RED_ | 0.96 |
| 5 | V_IP_ | 0.61 | V_IP_ | 0.78 | V_IP_ | 0.86 |
| 6 | φDo | -0.54 | ΔMR | 0.71 | ΔMR | 0.77 |
| 7 | δRo | 0.47 | δRo | 0.56 | RC/CSm | 0.74 |
| 8 | I_2_ | 0.42 | I_2_ | 0.55 | I_1_ | 0.73 |
| 9 | Vox | 0.28 | W_K_ | -0.55 | W_K_ | -0.73 |
| 10 | Ψ_O_ | 0.27 | I1 | 0.51 | V_ox_ | 0.73 |
| 11 | W_K_ | -0.24 | Ψ_O_ | 0.51 | I_2_ | 0.66 |
| 12 | I1 | 0.20 | RC/CSm | 0.47 | Ψ_O_ | 0.66 |
| 13 | φPo | 0.11 | Vox | 0.46 | δRo | 0.62 |
| 14 | W_L_ | -0.08 | φPo | 0.34 | φPo | 0.52 |
| 15 | RC/CSm | 0.05 | W_L_ | -0.23 | W_L_ | -0.30 |

Note: The negative value was marked in red. The data was obtained from the average data of wheat cultivars within different groups (G1, G2, and G3). n = 9, 14, and 14 for G1, G2, and G3, respectively.

**Table S2. Sensitivity of different parameters of wheat groups (G1, G2 and G3) related to G1**

| Parameters | Parameter values related to G1 | | |
| --- | --- | --- | --- |
|  | G1/G1 | G2/G1 | G3/G1 |
| φPo | 1.00 | 3.18 | 4.92 |
| ψ0 | 1.00 | 1.89 | 2.44 |
| δRo | 1.00 | 1.19 | 1.31 |
| φDo | 1.00 | 3.07 | 4.70 |
| Mo | 1.00 | 2.29 | 3.20 |
| RC/CSm | 1.00 | 9.45 | 14.77 |
| PI_tatal_ | 1.00 | 1.08 | 1.09 |
| V_IP_ | 1.00 | 1.27 | 1.41 |
| V_ox_ | 1.00 | 1.67 | 2.63 |
| V_RED_ | 1.00 | 1.30 | 1.36 |
| ΔMR | 1.00 | 1.11 | 1.20 |
| W_K_ | 1.00 | 2.30 | 3.06 |
| W_L_ | 1.00 | 3.01 | 3.90 |
| I_1_ | 1.00 | 2.62 | 3.70 |
| I_2_ | 1.00 | 1.32 | 1.58 |

Note:G1, G2, and G3 refer to measured data from cultivars in different groups.(n = 9, 14, and 14 for G1, G2, and G3, respectively).G1/G1=1 as a reference,G2/G1, G3/G1 means sensitivity of G2 and G3 relative G1.

**Table S3. The meaning of different parameters used in the manuscript.**

| Parameter | Parameter meaning |
| --- | --- |
| F_M_ | Maximum fluorescence intensity obtained under light after dark adaptation |
| F_O_ | Minimum fluorescence intensity (at 20 μs of PF curve) |
| Area | Total complementary area between the fluorescence induction curve and F = F_M_ |
| φP_o_ | Maximum quantum yield for primary photochemistry. |
| W_K_ | The activity of the donor side of photosystem II. |
| V_J_ | The relative variable fluorescence intensity at point J (2 ms). |
| V_I_ | The relative variable fluorescence intensity at point I (30 ms). |
| ψ_O_ | Efficiency/probability that an electron moves further than Q_A_^−^. |
| δR_o_ | Efficiency/probability with which an electron from the intersystem electron carriers is transferred to reduce end electron acceptors at the PSI acceptor side. |
| φE_o_ | Quantum yield for electron transport. |
| φD_o_ | Quantum yield for energy dissipation. |
| φR_o_ | Quantum yield for reduction of end electron acceptors at the PSI acceptor side. |
| M_o_ | Approximated initial slope (in ms^−1^) of the fluorescence transient normalized on the maximal variable fluorescence F_V_. |
| N | turnover number: number of Q_A_ reduction events between time 0 and t_（Fm）_ |
| RC/CS_m_ | Density of RCs (Q_A_-reducing PSII reaction centers) |
| PI_tatal_ | Performance index on absorption and transport basis |
| V_IP_ | Relative value of the IP segment of the fluorescence induction kinetic curve |
| W_L_ | The relative variable fluorescence intensity at point L (0.15 ms). |
| ΔMR | Maximum oxidation degree of PSI. |
| I_1_ | First peak of DF induction curve (at 7 ms) |
| I_2_ | Shoulder peak of DF induction curve (at 100 ms) |
