# Supplementary material for: Differential Sensitivity of Photosynthetic Electron Transport to Dark-Induced Senescence in Wheat Flag Leaves
Source: BMC Plant Biol. 2025 May 16;25:650. doi: 10.1186/s12870-025-06624-5 (PMC12082866; doi:10.1186/s12870-025-06624-5)
Supplement: Supplementary file 2 — Supplementary Material 2. [file 12870_2025_6624_MOESM2_ESM.docx]

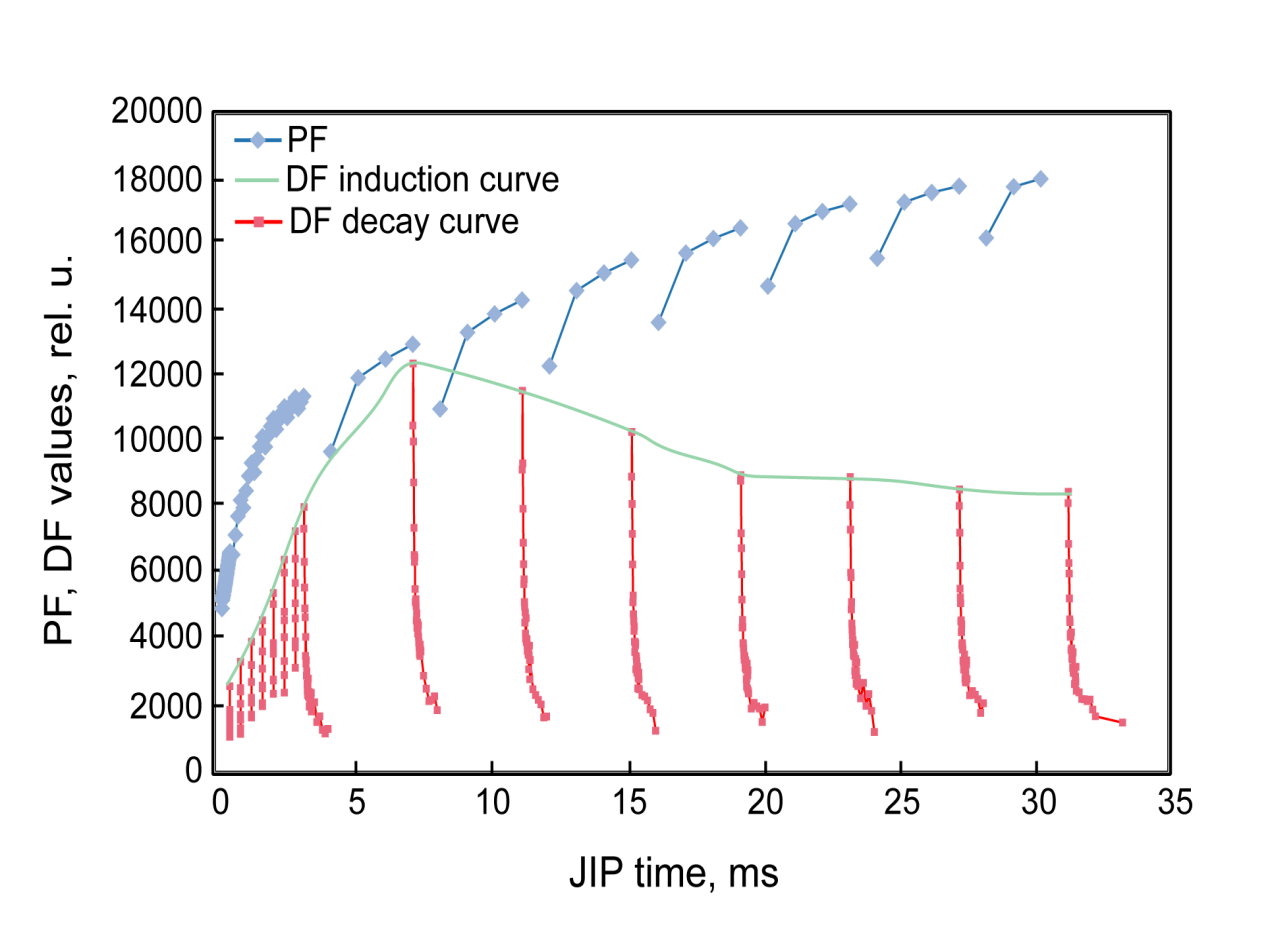


**Figure S1.** The diagrams of PF ,DF induction curve and DF decay curve in one measurement.

The prompt chlorophyll a fluorescence (PF) of the leaves increases following illumination,The DF induction curve construct by the DF signals are measured at the same point in the dark interval,

DF decay curve reflects the phenomenon where the DF intensity gradually decreases during each dark interval.


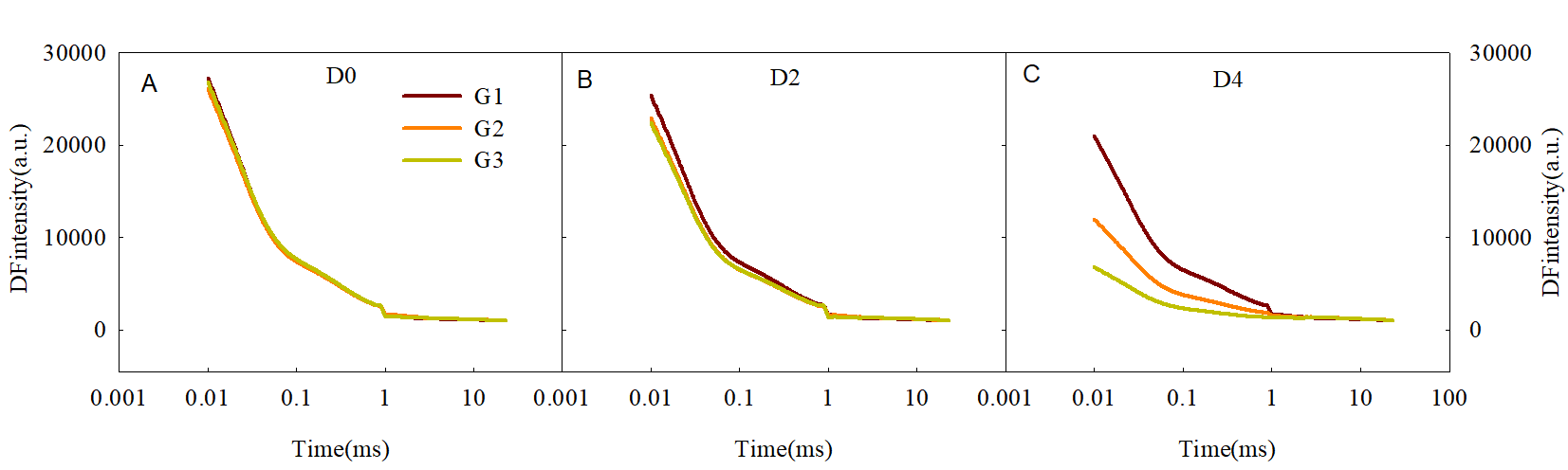


**Figure S2. DF decay curves at I1 in different cultivar groups treated for different time.** A: Before treatment; B: treated for 2 days;C:Treated for 4 days. Each curve was the average of all wheat cultivars in the corresponding group(G1, G2, and G3).
